# Interleukin-15 gene polymorphism in children with celiac disease: a single-center experience

**DOI:** 10.1007/s00431-025-06108-6

**Published:** 2025-04-23

**Authors:** Mohamed Abdelfadeel Ragab, Omneya M. Omar, Omneya Badreldin, Nahed Baddour, Hend A. Yassin, Nada M. Zeitoon

**Affiliations:** 1 Department of Pediatrics, Faculty of Medicine, Alexandria University, Alexandria, Egypt; 2 Department of Pathology, Faculty of Medicine, Alexandria University, Alexandria, Egypt; 3 Department of Medical Biochemistry, Faculty of Medicine, Alexandria University, Alexandria, Egypt

**Keywords:** Celiac disease, Anti-tissue transglutaminase immunoglobulin A, Serum interleukin-15, Interleukin-15 SNP, Pediatric age

## Abstract

The purpose of this study is to evaluate the demographics, clinical presentation, laboratory findings, and gastrointestinal endoscopic findings in children with CD and assess their relationship with interleukin-15 (IL-15) single-nucleotide polymorphism (SNP) (rs2857261) and serum IL-15 levels. This case–control and prospective cohort study included 54 newly diagnosed pediatric CD patients attending the Gastroenterology Clinic at Alexandria University Children’s Hospital and 44 age- and sex-matched healthy controls. Demographics, clinical data, laboratory tests, Marsh classification, and IL-15 SNP (rs2857261) genotypes were analyzed. Follow-up after 9 months on a gluten-free diet (GFD) was conducted. The mean age of patients and controls was 8.62 ± 4.4 and 8.07 ± 4.7 years, respectively, with no significant difference (*p* = 0.55). Male representation was 48.1% in patients and 47.7% in controls (*p* = 0.97). The most common presenting symptoms in CD patients were abdominal distension (61.11%) and failure to thrive (59.26%). Laboratory findings showed that mean anti-tissue transglutaminase immunoglobulin A was 103 ± 168 U/ml, and anti-endomysium immunoglobulin A was positive in 51.85% of patients. Histopathological assessment revealed Marsh 3C as the most common finding (37%), while 37% of patients were diagnosed without biopsy. IL-15 SNP (rs2857261) analysis showed a significantly higher prevalence of the A/A genotype in CD patients compared to controls (*p* < 0.0001). The A/G and G/G genotypes were protective against CD, with odds ratios of 0.088 and 0.079, respectively. No significant associations were observed between IL-15 genotypes and clinical, laboratory, or histological variables. After 6 to 9 months on a GFD, genotype did not significantly influence symptom resolution (*p* > 0.05).

*Conclusions*: Serum IL-15 levels are elevated in newly diagnosed pediatric CD patients. The IL-15 SNP (rs2857261) A/A genotype is associated with increased susceptibility to CD, while the A/G and G/G genotypes appear protective. These findings highlight IL-15 as a potential biomarker and therapeutic target in CD. Further large-scale studies are warranted to validate these findings and explore therapeutic applications.

**Table:** 

**What is Known:** • *Celiac disease is an immune-mediated enteropathy linked to HLA-DQ2/DQ8 alleles, with IL-15 playing a key role in its pathogenesis.* • *Variability in IL-15 genetic polymorphisms has been suggested but remains underexplored in pediatric populations.*
**What is New:** • *This study identifies the IL-15 SNP (rs2857261) A/A genotype as a risk factor for CD, while A/G and G/G genotypes are protective.* • *Elevated serum IL-15 levels in newly diagnosed patients highlight its potential as a biomarker and therapeutic target.*

**Supplementary Information:**

The online version contains supplementary material available at 10.1007/s00431-025-06108-6.

## Introduction

Celiac disease (CD) is a chronic gluten-induced, immune-mediated enteropathy with recent estimates of global prevalence around 1% of population [1, 2]. It is characterized by a variable combination of gluten-dependent clinical manifestations, CD-specific antibodies, and enteropathy [3, 4]. The disease occurs in individuals carrying the human leukocyte antigen (HLA) class II DQ2 or DQ8 allelic variants haplotype and is marked by an inflammatory enteropathy of varying severity, along with a wide range of gastrointestinal and extraintestinal symptoms and signs [5].

Celiac disease pathogenesis involves the passage of immunogenic gluten peptides through the intestinal epithelium, triggering adaptive and innate immune responses in the lamina propria. CD predominantly affects the duodenal intestine and induces a general flattening of the mucosa characterized by villous atrophy, crypt hyperplasia, and increased lymphocyte infiltration of the epithelium [6].

Diagnosis of CD is challenging due to its nonspecific and heterogeneous clinical presentation. Symptoms usually occur in children after ingestion of gluten-containing grains between 4 and 24 months of age. There may be a delay or latent period between gluten intake and the onset of symptoms [1, 7].

The symptoms can vary in intensity but commonly it presents with abdominal symptoms such as malabsorption, discomfort, constipation, loose stools, and flatulence and a variety of non-intestinal symptoms that include short stature, delayed puberty, anemia, liver abnormalities, joint and muscular disorders, neurological complications, psychiatric disorders, and cutaneous and mucosal manifestations. Importantly, CD can also present without symptoms and can only be diagnosed by screening [1, 8, 9].

Moreover, CD is strongly associated with autoimmune conditions such as type 1 diabetes mellitus and congenital disorders like immunoglobulin A deficiency and Down syndrome [10]. High-risk individuals, including first-degree relatives of CD patients and type 1 diabetics with suggestive signs, symptoms, or laboratory findings, should be screened [11].

Celiac disease is characterized by the presence of autoantibodies generated in response to gluten, which are highly specific to the condition. These antibodies have been extensively validated and are used for diagnostic and follow-up purposes. The two major autoantibodies are anti-tissue transglutaminase immunoglobulin A (anti-tTG IgA) and anti-endomysium immunoglobulin A (anti-EMA IgA) [12].

The management of CD requires strict adherence to a gluten-free diet (GFD), which can be challenging and expensive [13].

Adherence to a GFD is crucial not only for intestinal mucosal recovery and alleviation of symptoms, but also for the prevention of complications such as anemia, osteoporosis, fractures, and small bowel lymphoma [14].

Recent human genome studies have identified numerous non-HLA gene loci and specific single-nucleotide polymorphisms (SNP) related to cytokines involved in CD [15]. One of these cytokines is interleukin-15 (IL-15), which has a pivotal role in the immunopathogenesis of CD, as shown by significant positive correlations between IL-15 levels and histopathological severity of the disease [15, 16].

It is hypothesized that gliadin peptides indirectly enhance IL-15 expression by intraepithelial cells, primarily through the upregulation of inflammatory mediators resulting from T-cell activation. IL-15 is upregulated in both the epithelium and lamina propria, where it acts on various cell types and promotes immune dysregulation, contributing to CD pathogenesis [17].

Given its central role in CD pathogenesis, IL-15 represents a promising therapeutic target. Current treatments for CD are limited to a GFD, which is ineffective for many patients who progress to refractory CD. Targeting IL-15 could offer a novel approach to treating these patients, potentially reducing inflammation and preventing disease progression [18].

Evolving evidence highlights the crucial role of IL-15 in driving inflammation and tissue destruction in CD. Strategies to block IL-15 action could pave the way for personalized medicine [18]. The advent of biologic therapeutics has significantly improved outcomes in many autoimmune diseases, and similar advancements are anticipated for CD. Further studies are needed to evaluate the efficacy of IL-15-targeted therapies and to elucidate underlying mechanisms [19, 20].

In this study, we aimed to investigate the demographics, clinical presentation, laboratory findings, and gastrointestinal endoscopic findings of children with CD attending the Gastroenterology Clinic in our center. Additionally, we sought to assess the relationship between IL-15 SNP and serum IL-15 levels and their effect on disease severity.

## Materials and methods

This was a mixed case–control and prospective cohort study that included all newly diagnosed patients with CD attending the Gastroenterology Clinic in Alexandria University Children’s Hospital. The diagnosis of CD followed the European Society for Pediatric Gastroenterology, Hepatology, and Nutrition guidelines for CD diagnosis [21]. Healthy children of matched age and sex with no evidence of gastrointestinal diseases and no evidence of family history of CD were included as a control group.

Ninety-eight children were included in this study, 54 patients with CD diagnosis (Group I) and 44 healthy control subjects (Group II). A sample size of 88 children (44 per group) was needed to detect the difference in IL-15 gene SNP between CD patients (Group I) and healthy control group (Group II), assuming an effect size of 0.3 at 2 degrees of freedom, according to a previous study using a software by Hintze J (USA: NCSS, LLC., Kaysville, Utah; 2004) with a Pearson chi-square test at 0.05 level of significance [22].

The inclusion criteria were children from 1 year up to 18 years diagnosed with CD; they were not on a GFD, with the following additional inclusion criteria:

1. Positive celiac-specific serology and histopathological findings according to the Marsh classification.
2. Anti-tTG IgA levels more than 10 times the upper limit of normal, with positive anti-EMA IgA, with or without a biopsy.
3. In cases of immunoglobulin A deficiency, Marsh classification findings on biopsy were used [4].

Exclusion criteria included patients with acute or chronic gastrointestinal diseases other than CD, such as gastroenteritis, gastritis, inflammatory bowel disease, hepatitis, or cirrhosis, as well as patients with villous atrophy due to non-CD causes.

Written informed consent was obtained from all children’s parents or guardians.

The study was approved by the Ethical Committee of Alexandria University (IRB code 00012098‑FWA: No. 00018699. Ethics approval number: No. 0201709).

All patients underwent the following procedures:

I. A thorough history and clinical examination were conducted, focusing on the onset of symptoms, gastrointestinal symptoms, and extraintestinal symptoms. General and systemic examinations included a special emphasis on anthropometric measurements. *Z*-scores for body weight, height, and body mass index (BMI) were calculated using the World Health Organization (WHO) growth charts [23]. Signs of malnutrition, wasting, edema, arthritis, rash, and/or pallor were assessed. Abdominal examinations included abdominal distension, abdominal tenderness, organomegaly, ascites, or any palpable mass including fecal mass [24]. Follow-up after 9 months on a GFD was conducted.
II. Investigations done:
  1. **Blood sampling:** Five milliliters of venous blood was collected from the antecubital vein under aseptic conditions. The first blood fraction (3 ml) was collected in a clean centrifuge tube without anticoagulant to separate serum for biochemical analysis. Serum samples were centrifuged at 8000 rpm for 15 min. Hemolyzed samples were discarded. The second blood fraction (2 ml) was used for deoxyribonucleic acid (DNA) extraction and transferred into disposable plastic tubes containing ethylenediaminetetraacetic acid (EDTA). All samples were stored at − 20 °C [25, 26].d sampling 
  2. **Laboratory investigations: **for celiac patients included complete blood count, calcium profile (serum calcium, phosphorus, alkaline phosphatase), iron profile (serum iron and total iron binding capacity), and serum albumin. Celiac disease serology was performed, including total IgA, anti-tTG IgA (via the standard ELISA technique), and anti-EMA IgA [27]
  3. **Serum: **IL-15 was detected by Enzyme-Linked Immunosorbent Assay Kit provided by Chongqing Biospes Co, China, catalogue No.: BEK1122 according to the manufacturer’s directions. The IL-15 Quantitative Test Kit is based on sandwich enzyme-linked immune-sorbent assay technology. Duplicate readings for each standard, control, and sample were averaged, and the mean zero-standard optical density was subtracted. A standard curve was created and the mean absorbance value for each sample was used to determine the corresponding concentration of IL-15 in pg/ml [26].
  4. **IL-15 SNP genotyping assay (**28**, **29**):** Genotyping for IL-15 SNP (rs2857261); located on Chr.4:141719484 was done using TaqMan SNP Genotyping Assay. DNA was purified from whole blood samples using a spin column protocol with the QIAamp DNA Blood Mini Kit (Qiagen, Hilden, Germany). nanoDrop 2000 (Thermoscientific; USA) was used to check the quality and quantity of DNA samples [25]. IL-15 SNP (rs2857261) was genotyped using 40× TaqMan® Predesigned SNP Genotyping Assays (provided by Thermo Fisher Scientific, Waltham, MA, USA). The context sequence of IL-15 (rs2857261) SNP is TGAGTAATGAGAATTTCGGTAAGAA[A/G]AAAAATAGATGAAAATATCCTATGG. The A allele was detected with VIC® dye and the G allele with FAM™ dye. The 40× Predesigned SNP Genotyping Assay was diluted to a 20× working solution with nuclease-free water. The reaction mix was composed of 40× TaqMan® Genotyping Assay, TaqMan® Genotyping Master Mix, and nuclease-free water. The recommended final reaction volume per well was 20 µL for a 48-well plate (17 µL reaction mix + 3 µL DNA sample. To prepare the reaction mix, 10 µL of 2× TaqMan® Genotyping Master Mix, 1 µL of 20× Assay Working Solution (0.5 µL 40× TaqMan assay + 0.5 µL nuclease-free water), and 6 µL of nuclease-free water were added in each well. The total reaction volume uses 20 ng of genomic DNA. Real-time Polymerase Chain Reaction (PCR) was performed using Applied Biosystems StepOne™ Real-Time PCR System. In the real-time PCR system software, an experiment or plate document was using the following thermal Cycling Conditions; first AmpliTaq Gold® Enzyme Activation step at 95 °C for 10 min then 40 cycles; each consists of 15 s at 95 °C for denaturation and 1 min at 60 °C for annealing/extension [30].
  5. **Endoscopic evaluation and biopsy: **where histological analysis of duodenal biopsies with Marsh classification was done as well as counting of lymphocytes per high-power field, and morphology at the time of diagnosis. No biopsy approach was applied in patients with anti-tTG IgA more than 10 times the upper limit of normal and with positive anti-EMAIgA [21]. In the gastrointestinal (GI) endoscopy laboratory of our tertiary hospital, upper GI endoscopy was performed using a pediatric Olympus esophagogastroduodenoscope (type EVIIS EXERA II) under general anesthesia after preparation (no milk or solid food for 6 h prior to the scheduled procedure). After air insufflation, the macroscopic appearance of the mucosa was documented at the duodenal bulb and the second and third parts of the duodenum. Four mucosal samples were collected from the second part of the duodenum and one from the duodenal bulb. The specimens were immediately immersed in buffered formalin directly from the biopsy forceps. The severity of intestinal damage was graded by the pathologist as per the Marsh-Oberhuber classification.

### Statistical analysis

Statistical analyses were performed using IBM SPSS Statistics software, version 22.0 (IBM Corp., Armonk, NY, USA) [31]. The normality of data distribution was assessed using the Kolmogorov–Smirnov test. For normally distributed variables (except age), results were presented as arithmetic means and standard deviations (SD). Data for age were expressed as mean, median (minimum–maximum), and interquartile range (IQR). Categorical variables were compared using the chi-squared test or Fisher’s exact test when appropriate. The means of two non-normally distributed variables were compared using the Mann–Whitney *U* test. For comparing the means of nonparametric variables across more than two groups, the one-way ANOVA test was used. Logistic regression analysis was performed for variables showing significant differences between groups. Odds ratios (OR), their standard errors, and 95% confidence intervals (CI) were calculated following Altman’s method (1991). A *p* value of less than 0.05 was considered statistically significant. The genotype distribution of IL-15 polymorphisms in the control group was analyzed to verify conformity with the Hardy–Weinberg equilibrium [32, 33].

## Results

**Fig. 1:**
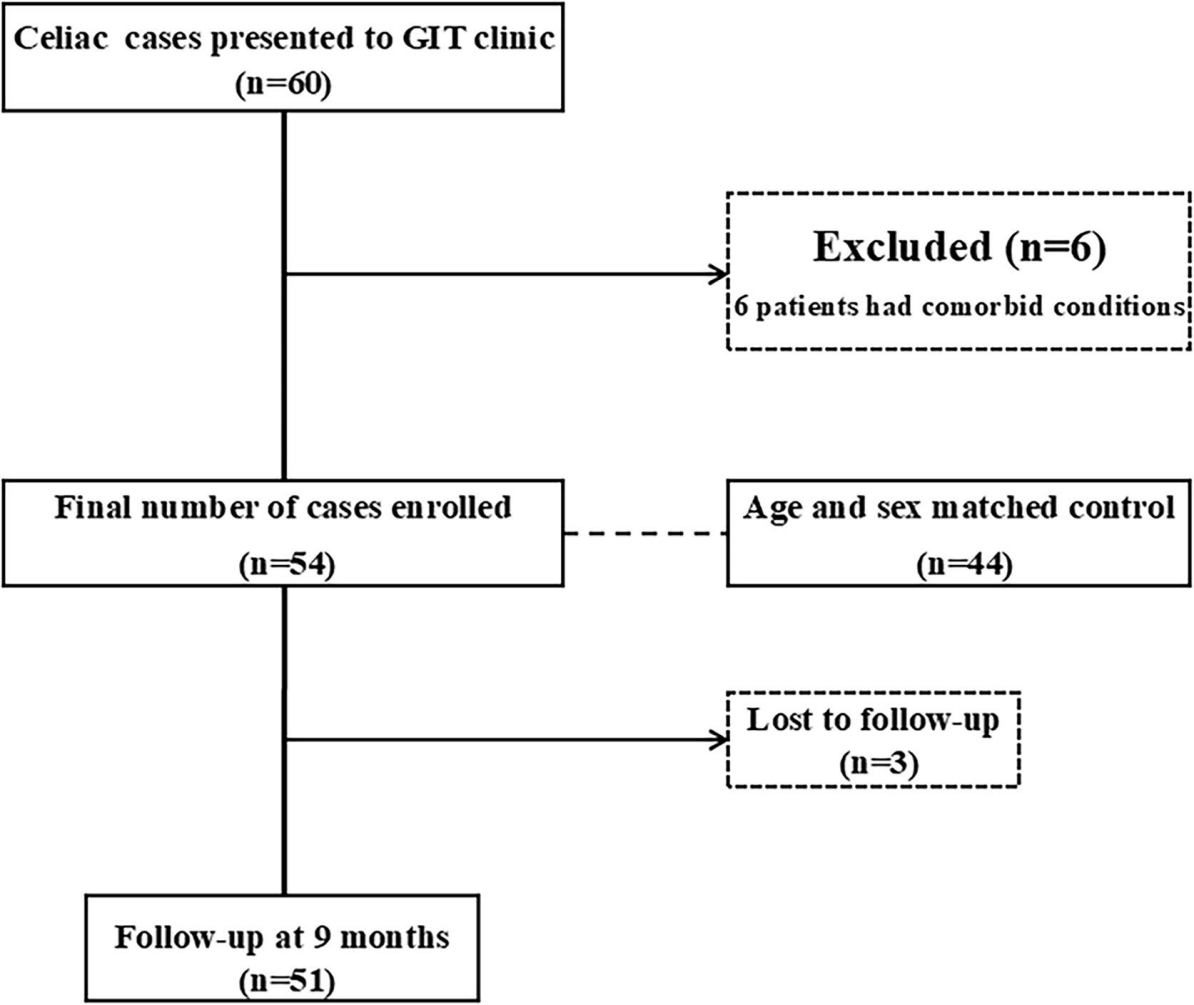
RECORD flow diagram of recruitment of the studied population

A total of 54 patients and 44 healthy controls were enrolled in the study. Follow-up was conducted 9 months after diagnosis and initiation of GFD. Of the initial cohort, 3 patients were lost to follow-up, leaving 51 patients for analysis (Fig. 1).

The mean age of cases was 8.62 ± 4.4 years, while for controls, it was 8.07 ± 4.7 years, with no significant difference observed (*p* = 0.55). The gender distribution was balanced, with 48.15% males in the case group and 47.72% females in the control group (*p* = 0.97), ensuring appropriate matching for age and gender.

The genotypic distribution of IL-15 SNP (rs2857261) revealed significant differences between cases and controls (*p* < 0.001). The AA genotype was found in 48.1% (26/54) of patients compared to only 6.8% (3/44) in controls. The AG genotype occurred in 35.2% (19/54) of patients and 56.8% (25/44) of controls, while the GG genotype was observed in 16.7% (9/54) of patients and 36.4% (16/44) of controls. Both the AG and GG genotypes showed a protective effect compared to the AA genotype, with odds ratios of 0.088 (95% CI = 0.025–0.306, *p* < 0.001) and 0.065 (95% CI = 0.018–0.237, *p* < 0.001), respectively. The genotype frequencies in the control group were consistent with the Hardy–Weinberg equilibrium (HW *p* = 0.108). For allele frequencies, allele A was represented in 71 cases (65.7%) and 31 controls (35.2%), while allele G was found in 37 cases (34.3%) and 57 controls (64.8%) (*p* < 0.001). When comparing between cases and control regarding IL-15 (rs2857261) SNP, (A/G) vs (A/A), the OR was 0.088 and (A/G + G/G) vs (A/A) OR was 0.079 (*p* < 0.0001). This indicates that the presence of the mutant allele (G) seems to be protective against developing CD (Table 1).

**Table 1:** Comparison of IL-15 SNP (rs2857261) genotype and allele distribution between celiac disease cases and controls

	Cases (*n* = 54) Number (%)	Control (*n* = 44) Number (%)	*c*^2^	*p*	OR (LL–UL 95%C.I)
**Il-15 SNP (rs2857261)**			20.210*	< 0.001*	
AA	26 (48.1)	3 (6.8)			1.000
AG	19 (35.2)	25 (56.8)			0.088 (0.023–0.333)^1^
GG	9 (16.7)	16 (36.4)			0.065 (0.015–0.276)^2^
^**HW**^**p**	**0.108**	**0.104**			
AA®	26 (48.1)	3 (6.8)	19.877*	< 0.001*	1.000
AG + GG	28 (51.9)	41 (93.2)			0.079 (0.022–0.286)
**Allele**			18.089*	< 0.001*	
A®	71 (65.7)	31 (35.2)			1.000
G	37 (34.3)	57 (64.8)			0.283 (0.157–0.512)

*c*^*2*^, chi-square test; *FE*, Fisher Exact; *OR*, odds ratio; *®*, reference group; *CI*, confidence interval; *LL*, lower limit; *SNP*, single-nucleotide polymorphism; *UL*, upper limit

*p*: *p* value for univariate regression analysis

^*^Statistically significant at *p* ≤ 0.05

^*HW*^*χ*^*2*^: chi-square for goodness of fit for the Hardy–Weinberg equilibrium (if *p* < 0.05—not consistent with HWE.)

^1^OR between AA and AG

^2^OR between AA and GG

Serum IL-15 levels were significantly elevated in the patient group, with a mean value of 15.45 ± 1.21 pg/ml, compared to 14.18 ± 0.15 pg/ml in controls (*p* < 0.001) (Table 2).

**Table 2:** Comparison between the two studied groups according to level of serum IL-15 (pg/ml)

	Cases (*n* = 54)	Control (*n* = 44)	*t*	*p*
**IL-15**				
Min.–max	14.30–20.30	13.90–14.50	6.900*	< 0.001*
Mean ± SD	15.45 ± 1.21	14.18 ± 0.15		
Median (IQR)	14.90 (14.70–15.50)	14.20 (14.10–14.25)		

*IQR*, inter quartile range; *SD*, standard deviation; *t*, Student’s *t*-test

*p*: *p* value for comparing the two studied groups

^*^Statistically significant at *p* ≤ 0.05

**Fig. 2:**
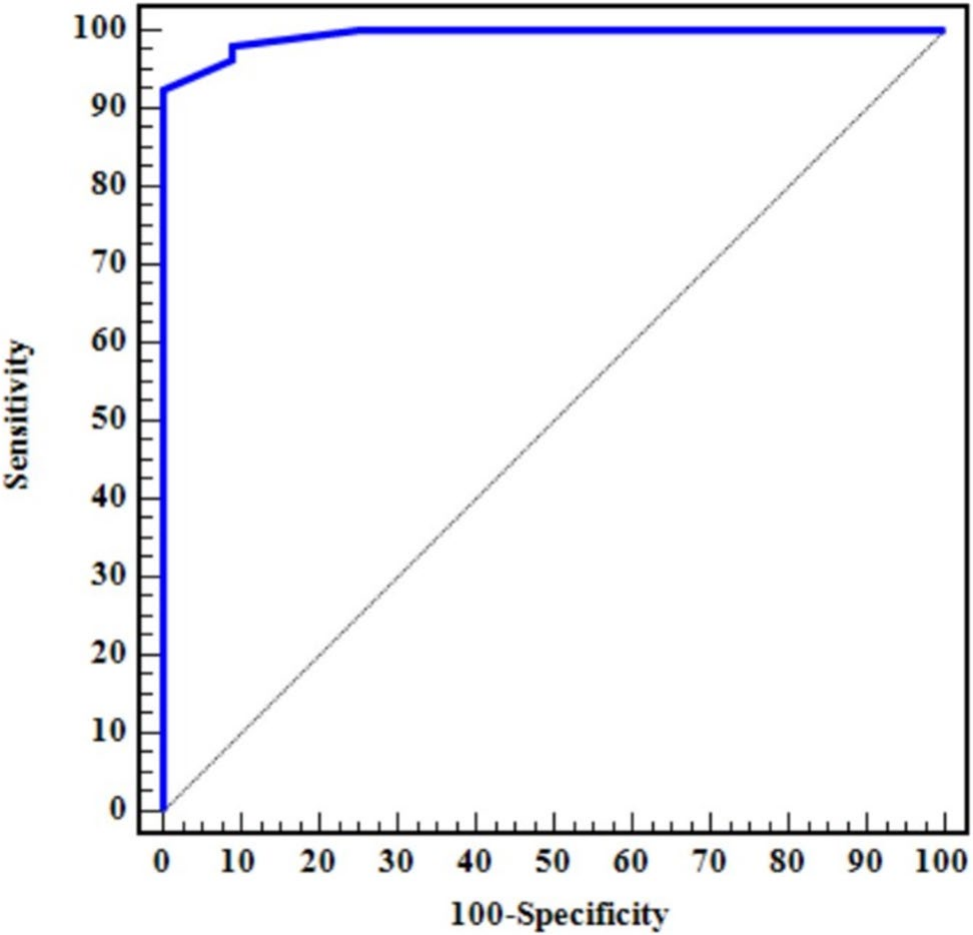
ROC curve analysis of IL-15 to differentiate celiac disease patients from controls

Receiver-operating characteristic (ROC) curve analysis demonstrated excellent diagnostic accuracy for IL-15 in distinguishing cases from controls, with an area under the curve (AUC) of 0.993 (*p* < 0.001). At a cut-off value of 14.3 pg/ml, IL-15 had a sensitivity of 98.15%, specificity of 90.91%, a positive predictive value (PPV) of 93%, and a negative predictive value (NPV) of 97.6% (Table 3, Fig. 2).

**Table 3:** Diagnostic performance for IL-15 to discriminate patients (*n* = 54) from control (*n* = 44)

	AUC	*p*	95% C.I	Cut-off	Sensitivity	Specificity	PPV	NPV
IL-15	0.993	< 0.001*	0.984–1.000	> 14.3	98.15	90.91	93.0	97.6

*AUC*, area under a curve; *p value*, probability value; *CI*, confidence intervals; *IL-15*, interleukin-15; *NPV*, negative predictive value; *PPV*, positive predictive value

^*^Statistically significant at *p* ≤ 0.05

**Fig. 3:**
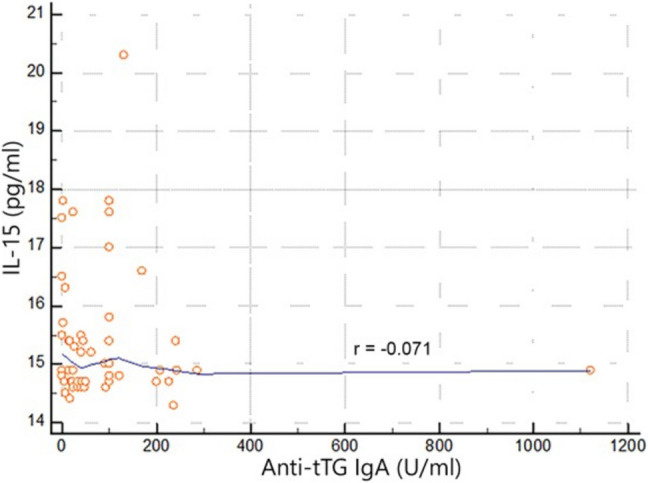
Correlation between serum IL-15 levels and anti-tTG IgA

There was no significant correlation between serum IL-15 level and anti-tTG IgA (Fig. 3).

Clinically, the most common presenting symptoms among patients included abdominal distention (61.11%), chronic diarrhea (44.4%), abdominal pain (57.4%), failure to thrive (59.3%), and chronic fatigue (55.6%). Associated autoimmune conditions were also noted, with 29.6% of patients having type 1 diabetes and 9.3% having thyroid disease (Table S1). Growth impairment was prominent, as 50% of patients had height-for-age below − 2 *Z* score, 40.74% had weight-for-age below − 2 *Z* score, and 14.81% had BMI-for-age below − 2 *Z* score (Table S2).

Laboratory investigations revealed anemia in 46.29% (25/54) of cases. Celiac disease serology showed elevated anti-tTG2 IgA levels (103 ± 168 U/ml); about 37% of patients had a level more than 100 U/ml and anti-EMA IgA positivity in 51.85% (28/54) of patients. The Marsh classification indicated varying degrees of intestinal damage, with Marsh 3C being the most common (37%), emphasizing the heterogeneity of mucosal involvement in the study population (Table 4).

**Table 4:** Investigations done for patients at the time of diagnosis (*n* = 54)

	*n* (%)
Hemoglobin (g/dl)	11.3 ± 1.9
Anemia (hemoglobin < 11.5 g/dl)	25 (46.29)
Serum albumin (g/dl)	3.93 ± 0.67
Serum calcium (mg/dl)	9.35 ± 0.96
Serum phosphorus (mg/dl)	4.4 ± 1.1
Alkaline phosphatase (IU/l)	280 ± 144
Serum iron (µg/dl)	45.7 ± 21
Total iron binding capacity (µg/dl)	337 ± 85
Total IgA (mg/dl)	145 ± 73
Anti-TTG2 IgA (U/ml)	103 ± 168
Anti-EMA (positive)	28 (51.85)
Marsh classification	
2	1 (1.9)
3A	10 (18.5)
3B	3 (5.6)
3C	20 (37)
Not done	20 (37)

*EMA*, endomyseal; *IgA*, immunoglobulin A; *IL*, interleukin; *TG2*, tranglutaminase-2; *SNP*, single-nucleotide polymorphism

**Fig. 4:**
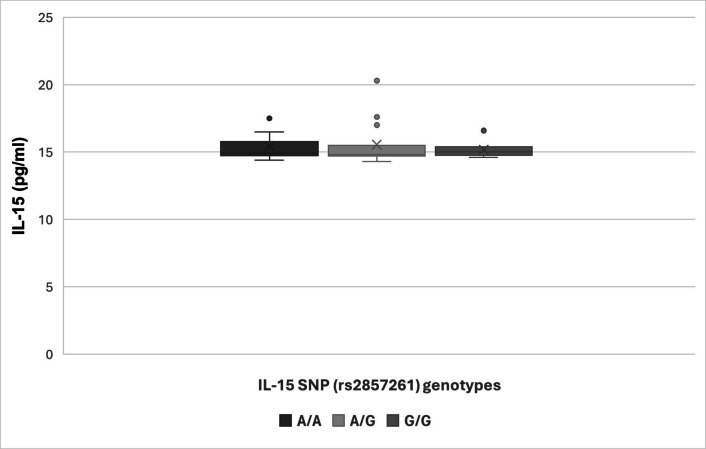
Graphical representations of IL-15 levels and genotype frequency distribution

Subgroup analysis of clinical and laboratory parameters by IL-15 genotype (AA, AG, GG) showed no significant differences across genotypes (*p* > 0.05). Growth parameters, hemoglobin levels, anti-tTG2 IgA, and IL-15 levels were similar among genotypes (Table 5, Fig. 4).

**Table 5:** Subgroup analysis according to IL-15 SNP genotypes

	A/A (*n* = 26) *n* (%)	A/G (*n* = 19) *n* (%)	G/G (*n* = 9) *n* (%)	*F*	*p*
**Age (years)**					
Min.–max	2–18	1–15	3–14		
Mean ± SD	8.35 ± 4.5	8.6 ± 4.7	9.4 ± 3.6	0.81	0.82
Median (IQR)	8 (4–12)	10 (6–12)	10 (6–12)		
**Gender (male)**	12 (46.2)	9 (47.4)	5 (55.6)	0.51	0.633
**Age of introduction of gluten (months)**					
Min.–max	4–14	3–6	5–6		
Mean ± SD	6.3 ± 1.8	5.6 ± 0.85	5.89 ± 0.33	0.32	0.235
Median (IQR)	6 (6–6)	6 (6–6)	6 (6–6)		
**Symptoms appearance (years)**					
Min.–max	1–17	1–13	2–14		
Mean ± SD	6.8 ± 4.7	7.1 ± 4.2	7.78 ± 4.6	0.06	0.91
Median (IQR)	7 (4–10)	7.5 (4–10)	9 (4–12)		
**Consanguinity (positive)**	7 (26.9)	4 (21.1)	3 (33.3)	0.43	0.49
**Family history of celiac disease**	4 (15.4)	4 (21.1)	2 (22.2)	0.52	0.65
**Family history of other autoimmune disease**	4 (15.4)	3 (15.8)	1 (11.1)	0.32	0.75
Chronic diarrhea	9 (34.6)	11 (57.9)	4 (44.4)	2.81	0.12
Chronic abdominal pain	15 (57.7)	12 (63.2)	4 (44.4)	0.21	0.36
Abdominal distension	17 (65.4)	11 (57.9)	5 (55.6)	0.56	0.61
Constipation	6 (23.1)	7 (36.8)	4 (44.4)	1.4	0.23
Recurrent nausea and vomiting	8 (30.8)	5 (26.3)	2 (22.2)	0.57	0.63
Failure to thrive	15 (57.7)	12 (63.2)	5 (55.6)	0.69	0.71
Delayed puberty	2 (7.7)	3 (15.8)	1 (11.1)	0.15	0.4
Chronic fatigue	15 (57.7)	12 (63.2)	3 (33.3)	2.25	0.15
Resistant iron deficiency anemia	7 (26.9)	3 (15.8)	2 (22.2)	0.19	0.38
Unexplained elevated liver functional tests	1 (3.8)	1 (5.3)	0 (0)	0.16	0.49
Unexplained arthritis or arthralgia	9 (34.6)	5 (26.3)	2 (22.2)	0.45	0.5
Short stature	13 (50)	10 (52.6)	4 (44.4)	0.51	0.69
Recurrent aphthous stomatitis	5 (19.2)	3 (15.8)	1 (11.1)	0.49	0.58
Dermatitis herpetiformis-type rash	4 (15.4)	0 (0)	0 (0)	1.41	0.22
Dental enamel defects	12 (46.2)	7 (36.8)	4 (44.4)	0.69	0.53
**Signs of malnutrition**					
Underweight	16 (61.5)	10 (52.6)	5 (55.6)	0.65	0.56
Muscle wasting	12 (46.2)	8 (42.1)	4 (44.4)	0.43	0.79
Lower limb edema	3 (11.5)	1 (5.3)	0 (0)	0.19	0.29
Signs of vitamins deficiency	11 (42.3)	10 (52.6)	4 (44.4)	0.65	0.5
Features of anemia	10 (38.5)	8 (42.1)	4 (44.4)	0.42	0.76
**Laboratory data**					
Hemoglobin (g/dl)	11.6 ± 2.1	11 ± 1.9	11.5 ± 1.1	0.57	0.64
Anemia (hemoglobin < 11.5 g/dl)	10 (40)	12 (63.2)	3 (33.3)	0.17	0.34
Serum albumin (g/dl)	4 ± 0.8	3.74 ± 0.6	4 ± 0.4	0.15	0.35
Serum calcium (mg/dl)	9.33 ± 1.14	9.27 ± 0.83	9.6 ± 0.7	0.42	0.76
Serum phosphorus (mg/dl)	4.6 ± 1.38	4.11 ± 0.74	4.5 ± 0.8	0.15	0.43
Alkaline phosphatase (IU/l)	276 ± 169	262 ± 127	333 ± 101	0.66	0.52
Serum iron (µg/dl)	44 ± 18.6	47 ± 23.3	47 ± 24.4	0.05	0.91
Total iron binding capacity (µg/dl)	321 ± 68	347 ± 104	362 ± 89	0.16	0.44
Total IgA (mg/dl)	131 ± 58	164 ± 97	144 ± 47	0.15	0.36
Anti-TTG2 IgA (U/ml)	65 ± 84	143 ± 248	56 ± 55	1.49	0.24
Anti-EMA (positive)	12 (46.2)	12 (63.2)	4 (44.4)	0.15	0.36
Serum IL-15 (pg/ml)	15.4 ± 1	15.6 ± 1.6	15.2 ± 0.6	0.47	0.746
March classification					
2	1 (3.8)	0 (0)	0 (0)	0.59	0.56
3A	4 (15.4)	3 (15.8)	3 (33.3)	1.51	0.25
3B	3 (11.5)	0 (0)	0 (0)	1.37	0.29
3C	6 (23.1)	9 (47.4)	5 (55.6)	8.75	0.075
Not done	10 (38.5)	7 (36.8)	1 (11.1)	2.65	0.13

*EMA*, endomyseal; *IgA*, immunoglobulin A; *IL-15*, interleukin-15; *SNP*, single-nucleotide polymorphism; *TG2*, tranglutaminase-2

*F* for one-way ANOVA test that was used to compare means and proportions of the three groups

*p*: *p* value for comparing between the studied groups

^*^Statistically significant at *p* ≤ 0.05

Table S3 compares the distribution of IL-15 genotypes among patients with and without residual symptoms after follow-up. The A/A genotype was slightly more prevalent in patients with residual symptoms (50%) compared to those without residual symptoms (43.5%). Similarly, the A/G genotype showed comparable proportions in both groups (34.8% vs. 35.7%), while the G/G genotype was observed in 21.7% of patients without residual symptoms and 14.3% of those with residual symptoms.

Table S4 explores the relationship between IL-15 SNP genotypes and anti-tTG IgA levels after follow-up. Among patients with normal anti-tTG IgA levels, the A/A genotype was most frequent (48.7%), followed by the A/G (29.7%) and G/G (21.6%) genotypes. In patients with persistently high anti-tTG IgA levels, the A/A genotype was present in 42.9%, A/G in 50%, and G/G in 7.1%.

Statistical analysis revealed no significant associations between IL-15 genotypes and the persistence of symptoms or the normalization of anti-tTG IgA levels (*p* > 0.05 for all comparisons). These findings suggest that IL-15 genotype may not directly influence symptom resolution or serological response to treatment during the follow-up period.

## Discussion

In our prospective study, the mean age of participants was 8.62 ± 4.4 years, with a median age of 9 years. Females constituted a slight majority, accounting for 51.9% of the study population. Bardella et al. (2005) investigated gender- and age-related symptoms of gluten intolerance in a cohort of 1436 patients and found a higher prevalence among females (69.4%) [34]. Similarly, a more recent study by Kara et al. (2021) involving 90 patients with CD reported a median age of 11 years (range = 3–18 years) and a female predominance (54.4%) [29].

In our study, patients exhibited a wide range of clinical features. The most common presenting symptom was abdominal distention (61.11%), followed by failure to thrive (59.26%), chronic abdominal pain (57.41%), and chronic fatigue (55.56%). Similarly, Kara et al. (2021) reported that the most common symptom in their cohort was abdominal pain (52%), followed by weight loss (42%) and malnutrition (28%) (29). In a cross-sectional study conducted by Dehbozorgi et al. (2020) involving 130 patients with CD, abdominal pain was also the most prevalent symptom (62%), followed by bone pain (53%) and chronic fatigue (49%) [35].

A study from Saudi Arabia by Al Sarkhi et al. (2016), which included 113 patients, revealed that the most common presentation of CD was chronic abdominal pain (59%), followed by poor weight gain (54%) and abdominal distention (46%) [36]. Similarly, an older study by Poddar et al. (2006) involving 300 confirmed CD patients reported that the most frequent presenting symptoms were failure to thrive (91%), wasting (87%), anemia (84%), and diarrhea (84%) [37]. These findings highlight the vast variability in the clinical manifestations of CD, which can present with a wide range of gastrointestinal and extra-gastrointestinal symptoms.

In the current study, we observed significantly elevated serum IL-15 levels in newly diagnosed CD patients compared to the control group. Aghamohamadi et al. found that IL-15 messenger ribonucleic acid levels were notably higher in CD patients with Marsh II lesions compared to healthy controls, as well as those with Marsh I and Marsh III lesions. The difference between Marsh II and Marsh I patients was statistically significant. While serum IL-15 concentrations were also elevated in Marsh II patients compared to Marsh I and Marsh III patients, these differences did not reach statistical significance [38]. However, the study suggested that tissue levels of IL-15 correlate with circulating IL-15 expression. This supports the potential of circulating IL-15 as a reliable biomarker for assessing intestinal damage in CD [38].

IL-15 overexpression was also demonstrated in patients with active CD in the study by Mention et al. [39]. Numerous studies have shown that the gluten-driven intestinal inflammatory disorder in CD is primarily caused by the upregulation of IL-15 expression in the intestinal mucosa. IL-15 acts on various cell types and influences distinct immune components and pathways, ultimately disrupting intestinal immune homeostasis [17].

In our study, the diagnostic power of IL-15 was evaluated, demonstrating its potential as a promising tool for detecting CD with a sensitivity of 98.15% in the cases group. Furthermore, a serum level of IL-15 exceeding 14.3 pg/ml could be considered evidence of CD when compared to healthy controls.

An Iranian study by Masaebi et al. assessed the diagnostic performance of various cytokines for CD and non-celiac gluten sensitivity (NCGS). Their findings, for the first time, highlighted that IL-8 and IL-15 exhibited the highest sensitivities, specificities, and predictive values (positive and negative) for detecting CD patients compared to the NCGS group and healthy controls [40].

Similarly, a study conducted by Di Sabatino et al. evaluated 46 CD patients and 22 healthy individuals. Their results indicated that IL-15 expression significantly increased in the intestinal tissues of CD patients compared to healthy individuals. This finding suggests that a lower immunological threshold of IL-15 in CD contributes to the initiation of other immune responses and the development of small bowel lesions [41].

Bernardo et al. obtained similar findings after evaluating 42 CD patients and 24 healthy individuals at a gastroenterology clinic for intestinal pathologies. However, they noted that IL-15 levels did not increase in the NCGS group [42].

In another study by Heydari et al., they enrolled 110 treated CD patients, 15 with NCGS, and 46 healthy children, despite the higher mean serum levels of IL-15 they reported in the CD patients group (69.4 ± 137.9) as compared with patients in the NCGS (27.9 ± 61.1) and control (17.0 ± 43.9) groups, these differences were not significantly different between the studied populations (*p* = 0.869) [43].

Additionally, a study by Escudero-Hernandez et al. analyzed the IL-15 and IL-15Rα genes in samples from the Spanish Consortium for Genetics of Celiac Disease. Their findings suggested that the IL-15 gene might contribute to the genetic predisposition to CD and refractory CD through less common variants with moderate effects. They identified two regulatory SNP associated with CD: rs4956400 (*p* = 0.0112, OR 1.21, 95% CI 1.04–1.40) and rs11100722 (*p* = 0.0087, OR 1.24, 95% CI 1.06–1.45), both located upstream of the IL-15 gene. These SNP were found to correlate with higher IL-15 protein expression [16]

In our study, comparing the IL-15 SNP (rs2857261) between the patient and control groups, we found that the A/A genotype was significantly more prevalent among the patients (48.15%) compared to the control group (6.8%) with statistical significance (*p* < 0.0001). In contrast, the A/G genotype was more frequent in the control group (56.8%) than in the patient group (35.2%) with a significant difference (*p* < 0.0001). These findings contradict those of Kara et al., who studied Turkish patients with CD and found a significantly higher frequency of the GG genotype in celiac patients compared to controls. In their study, the AA genotype was more common in the control group than in the celiac patients [29].

Furthermore, we analyzed the potential impact of this IL-15 SNP (rs2857261) on CD symptoms and histopathological grade, but found that none of the clinical symptoms or the histological grade was influenced by the presence of any of the genotypes of this SNP. These discrepancies in clinical manifestations may be explained by the presence of additional modifier genes that may influence the disease phenotype."

In addition, differences in genotype findings could be attributed to variations in genetic backgrounds, as population-specific allele frequencies can significantly influence results [44]. The presence of modifier genes and genetic linkage with other variants may further explain population-specific effects [45].

Currently, limited data exist regarding IL-15 gene polymorphisms and their variability among individuals from different ethnic backgrounds and diseases. However, IL-15 gene variants have been associated with several autoimmune disorders, including psoriasis, type 1 diabetes, ulcerative colitis, and rheumatoid arthritis. These findings highlight the importance of genetic studies investigating the involvement of the IL-15 gene in celiac disease (CD) [16, 17].

Given the central role of IL-15 in the immunopathogenesis of CD, there is growing interest in developing novel therapies to attenuate its actions. To inhibit IL-15 activity and prevent its harmful effects on oral tolerance and intraepithelial lymphocyte activation, several therapeutic agents have been developed, including IL-15-specific antibodies [17].

While HLA genes are a prerequisite for the development of CD, they are not sufficient on their own. Approximately 39% of the general population carries the DQ2 or DQ8 genes, yet only 3% of these individuals develop CD. This highlights the role of additional genetic factors in determining host susceptibility to the disease [46].

This study has several limitations. Its single-center design may limit the generalizability of the findings. Additionally, serum and tissue IL-15 levels were not assessed after GFD adherence, particularly in patients with the A/A genotype, restricting insights into IL-15 expression dynamics. Resource constraints prevented the analysis of additional IL-15 SNPs beyond rs2857261. Future large-scale studies should evaluate IL-15 levels before and after GFD adherence, especially in genetically predisposed individuals, and investigate multiple IL-15 SNP to better understand IL-15’s role in CD and its therapeutic potential.

## Conclusion

This study revealed that the most common gastrointestinal symptoms in children with CD were abdominal pain, abdominal distension, and failure to thrive. The most frequent extraintestinal manifestations included failure to thrive, long-term fatigue, and short stature. Additionally, type 1 diabetes mellitus and hypothyroidism were identified as the most common comorbidities associated with CD.

Our findings demonstrated that IL-15 levels were elevated in newly diagnosed children with CD. Moreover, the study suggests that the IL-15 gene (rs2857261) AA variant is more prevalent among celiac patients compared to healthy controls, whereas the A/G and G/G variants appear to confer a protective effect against the disease.

This conclusion underscores the importance of IL-15 as a potential biomarker and therapeutic target in the management of CD.

## Supplementary Information

Below is the link to the electronic supplementary material.

Supplementary file1 (DOCX 20 KB)

## Data Availability

Data can be shared by the corresponding author with a reasonable request.

## Abbreviations

Anti-EMA IgA: Anti-endomysium immunoglobulin A
Anti-tTG IgA: Anti-tissue transglutaminase immunoglobulin A
AUC: Area under the curve
BMI: Body mass index
CD: Celiac disease
DNA: Deoxyribonucleic acid
EDTA: Ethylenediamine tetra acetic acid
GFD: Gluten-free diet
GI: Gastrointestinal
HLA: Human leucocytic antigen
IgA: Immunoglobulin A
IL-15: Interleukin-15
IQR: Interquartile range
NCGS: Non-celiac gluten sensitivity
NPV: Negative predictive value
OR: Odds ratio
PCR: Polymerase chain reaction
PPV: Positive predictive value
ROC: Receiver-operating characteristic
SD: Standard deviation
SNP: Single-nucleotide polymorphisms
WHO: World Health Organization
