# Supplementary material for: Interleukin-15 gene polymorphism in children with celiac disease: a single-center experience
Source: Eur J Pediatr. 2025 Apr 23;184(5):308. doi: 10.1007/s00431-025-06108-6 (PMC12018617; doi:10.1007/s00431-025-06108-6)
Supplement: Supplementary file 1 — Supplementary file1 (DOCX 20 KB) [file 431_2025_6108_MOESM1_ESM.docx]

**Table S1 Descriptive data of patients at the time of diagnosis (n = 54)**

|  | | n(%) |
| --- | --- | --- |
| **Symptoms appearance (years)** | | |
| Min. – Max. | | 1 – 17 |
| Mean ± SD. | | 7.06±4.4 |
| Median (IQR) | | 7 (3 – 11) |
| **Consanguinity (Positive)** | | 14 (25.9) |
| **Family history of celiac disease** | | 10 (18.5) |
| **Family history of other autoimmune disease** | | 8 (14.8) |
| **Symptoms** | |  |
| Chronic Diarrhea | | 24 (44.4) |
| Chronic abdominal pain | | 31 (57.4) |
| Abdominal distension | | 33 (61.1) |
| Constipation | | 17 (31.5) |
| Recurrent nausea and Vomiting | | 15 (27.8) |
| Failure to thrive | | 32 (59.3) |
| Delayed puberty | | 6 (11.1) |
| Chronic fatigue | | 30 (55.6) |
| Resistant iron deficiency anemia | | 12 (12.2) |
| Unexplained elevated liver functional tests | | 2 (3.7) |
| Unexplained arthritis or arthralgia | | 16 (29.6) |
| Short stature | | 27 (50) |
| Recurrent aphthous stomatitis | | 9 (16.7) |
| Dermatitis herpetiformis-type rash | | 4 (7.4) |
| Dental enamel defects | | 23 (42.6) |
| **Associated endocrinal and other diseases** | | |
| Type 1 diabetes mellitus | 16 (29.6) | |
| Thyroid disease | 5 (9.3) | |
| Down syndrome | 1 (1.9) | |
| Turner syndrome | 2 (3.7) | |
| Williams syndrome | 0 (0) | |
| Presence of selective IgA deficiency | 2 (3.7) | |

**Table S2 Anthropometric measurements of patients at the time of diagnosis**

|  | **Cases (n = 54)** |
| --- | --- |
| **Height /length for age Z score at diagnosis** | |
| Mean ± SD | -2.13±1.7 |
| Median (IQR) | -2 (-3 - -0.82) |
| Less than -2 Z score n(%) | 27 (50) |
| **Weight for age Z score at diagnosis** | |
| Mean ± SD | -1.55±1.62 |
| Median (IQR) | -1.7 (-2.49 - -0.44) |
| Less than -2 Z score n(%) | 22 (40.74) |
| **Body mass index for age Z score at diagnosis** | |
| Mean ± SD | -0.5±1.8 |
| Median (IQR) | -0.68 (-1.54 – 0.59) |
| Less than -2 Z score n(%) | 8 (14.81) |

**Table S3 Distribution of IL-15 SNP Genotypes among Patients with and without Residual Symptoms after Follow-Up**

|  | No residual Symptoms  (N = 23)  **n (%)** | | Residual Symptoms  (N = 28)  **n (%)** | | Test | P |
| --- | --- | --- | --- | --- | --- | --- |
| A/A | | 10 (43.5) | 14 (50) | χ² = 0.21 | | 0.65 |
| A/G | | 8 (34.8) | 10 (35.7) | χ² = 0.039 | | 0.84 |
| G/G | | 5 (21.7) | 4 (14.3) | χ² = 0.467 | | 0.49 |

χ^2^: **Chi square test**

p: p value for comparing between the studied groups

*: Statistically significant at p ≤ 0.05

**Table S4 Association between IL-15 SNP Genotypes and Anti-TTG IgA Levels on Follow-Up**

|  | Normal Anti-TTG IgA  (n = 37)  **n (%)** | High Anti-TTG IgA  (n = 14)  **n (%)** | Test | P |
| --- | --- | --- | --- | --- |
| A/A | 18 (48.7) | 6 (42.9) | χ² = 0.134 | 0.71 |
| A/G | 11 (29.7) | 7 (50) | χ² = 1.797 | 0.18 |
| G/G | 8 (21.6) | 1 (7.1) | χ² = 1.442 | 0.23 |

χ^2^: **Chi square test**

p: p value for comparing between the studied groups

*: Statistically significant at p ≤ 0.05
